# Factors Affecting the Quality of Non-contrast Coronary Magnetic Resonance Angiography Images: Challenges and Change

**DOI:** 10.31083/RCM37487

**Published:** 2025-08-19

**Authors:** Wei Deng, Shichu Liang, Feidan Yu, Caiyun Han, Hong Ren

**Affiliations:** ^1^Department of Radiology, Sir Run Run Shaw Hospital, Zhejiang University School of Medicine, 310016 Hangzhou, Zhejiang, China; ^2^Department of Cardiology, West China Hospital, Sichuan University, 610041 Chengdu, Sichuan, China; ^3^Department of Radiology, First Affiliated Hospital of Anhui Medical University, 230032 Hefei, Anhui, China

**Keywords:** coronary magnetic resonance angiography, coronary artery disease, imaging quality, influencing factors

## Abstract

Cardiovascular diseases (CVDs) are the main cause of mortality worldwide, with coronary artery disease (CAD) noted as one of the major causes of CVD. An early and accurate diagnosis is important for improved outcomes in CAD patients. Invasive coronary angiography and coronary computed tomography angiography are accurate diagnostic tools for CAD. However, these examination methods possess limitations, including invasiveness and use of ionizing radiation, which limit their application in certain population groups. Meanwhile, coronary magnetic resonance angiography (CMRA) represents a noninvasive method that provides high-resolution coronary artery images without ionizing radiation and contrast agents. Nonetheless, the quality of CMRA images depends on numerous physiological and technical factors. This review analyzes the main factors that affect CMRA image quality and provides theoretical and technical insights for better clinical application of CMRA in CAD diagnoses.

## 1. Introduction

Cardiovascular diseases (CVDs) are the leading cause of mortality worldwide, affecting over 523 million people. The early diagnosis of coronary artery disease (CAD), which forms the major component of CVD, is important for the prognosis of patients [1, 2]. Invasive coronary angiography (ICA) is the “gold standard” for diagnosing CAD [3]. However, the invasive nature of ICA render this method unsuitable for routine CAD screening. Meanwhile, coronary computed tomography angiography (CCTA) comprehensively assesses coronary artery stenosis, fractional flow reserve, and plaque morphology, making CCTA the most commonly utilized noninvasive imaging modality for CAD diagnosis [4]. However, ionizing radiation has limited the application of CCTA in certain populations, such as young patients who require repeated imaging, pregnant women, and those needing regular follow-up examinations (Table 1) [5].

**Table 1.** **Comparison between CCTA and CMRA**.

Comparison item	CCTA	CMRA
Radiation dose	3–8 mSv (X-ray ionizing radiation)	0 (no ionizing radiation)
Contrast agent usage	Requires iodinated contrast (allergy risk)	Routine sequences require no contrast (gadolinium needed for some sequences)
Temporal resolution	75–200 ms	Flexible (∼30 ms)
Spatial resolution	0.5 mm	1 mm
Imaging time	<10 seconds	>5 minutes
Operator dependency	Low (standardized protocols)	High (parameter optimization required)
Sensitivity	97% (95% CI: 96.2%–98.0%)	86% (95% CI: 80%–90%)
Specificity	87% (95% CI: 84.5%–89.9%)	73% (95% CI: 65%–81%)
Cost-effectiveness	Lower cost	Higher cost
Diagnostic performance	High spatial resolution, superior coronary anatomy visualization; limited by calcification/stent artifacts	Excellent soft-tissue contrast, strong functional assessment; lower resolution for distal coronaries

Note: CCTA, coronary computed tomography angiography; CMRA, coronary magnetic resonance angiography; CI, confidence interval.

**Fig. 1.**
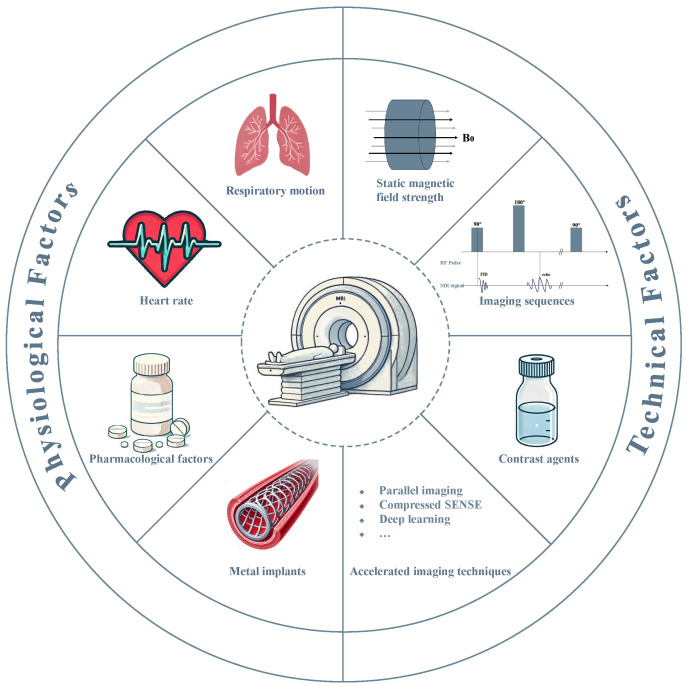
**Fundamental determinants on the quality and clinical utility of CMRA**. MR, magnetic resonance; SENSE, sensitivity encoding; RF, radio frequency; FID, free induction decay.

Conversely, coronary magnetic resonance angiography (CMRA) represents an advanced noninvasive imaging modality for screening and longitudinal CAD assessment, primarily due to its non-ionizing nature and the obviation of exogenous contrast agents [6]. Previous studies have demonstrated that CMRA is important in diagnosing and assessing CAD [7, 8, 9, 10], particularly in identifying coronary artery stenosis and evaluating vessel wall characteristics. However, the utilization of CMRA is predicated through exceptionally high image quality, which is inherently influenced by multiple factors, including physiological conditions (e.g., respiratory motion, cardiac pulsation, metal implants, pharmacological factors), and technical imaging parameters (e.g., magnetic field strength, pulse sequences, contrast agent protocols) (Fig. 1). This review discusses the key factors that influence CMRA image quality, providing theoretical and technical insights to enhance its clinical applicability in CAD diagnoses.

## 2. General Technical Principles of CMRA

The working principle of CMRA involves three main stages: polarization and radiofrequency pulse application, motion compensation, and signal processing and image reconstruction.

### 2.1 Polarization and Radiofrequency Pulse Application

Hydrogen nuclei within the body are polarized by applying a strong external magnetic field, causing them to align along a defined axis. Upon introducing a radiofrequency pulse, typically employing a gradient echo sequence or steady-state free precession sequence, the magnetic moments of these hydrogen nuclei enter a resonant state, deviating from their equilibrium configuration. Following the cessation of the radiofrequency pulse, the nuclei gradually return to equilibrium, emitting radiofrequency signals that are subsequently detected by receiver coils to produce diagnostic images. The spin–lattice relaxation time (T1) and spin–spin relaxation time (T2) are critical parameters that influence the resultant image contrast and resolution. Therefore, by refining pulse sequences, the contrast between different tissue types can be significantly enhanced, augmenting the diagnostic precision of coronary imaging.

### 2.2 Cardiac and Respiratory Motion Compensation

Cardiac motion represents one of the major sources of motion in coronary artery images. CMRA commonly incorporates electrocardiogram (ECG) gating and respiratory gating to reduce the effects of cardiac and respiratory motion. ECG gating collects data at specific phases in the cardiac cycle, typically at end-diastole, where cardiac motion is minimal, to reduce motion artifacts. Meanwhile, respiratory gating can synchronize image acquisition to reduce motion artifacts associated with respiration. Recent improvements in image reconstruction algorithms for free-breathing conditions have allowed high-quality coronary imaging without patients needing to hold their breath, thus improving patient comfort and feasibility.

### 2.3 Signal Processing and Image Reconstruction

The acquired radiofrequency signal is digitized and processed by a Fourier transform to produce spatial domain images. The Fourier transform is a required computational step that enables the signals acquired in the time domain to be converted to the frequency domain so that the magnetic resonance imaging (MRI) signal frequency and phase characteristics can be accurately mapped to spatial coordinates, where spatial images must be reconstructed. This step is essential for reconstructing high-resolution spatial images, which are needed to visualize the anatomy of the heart and coronary arteries [11, 12].

## 3. Physiological Factors on Imaging Quality

### 3.1 Respiratory Motion and Compensation Strategies

Respiratory motion is one of the main sources of image quality degradation in CMRA. Thoracic displacement during respiration induces complex spatial shifts and morphological alterations in coronary arteries, leading to significant motion artifacts that compromise spatial resolution and diagnostic accuracy in imaging. Recent advancements in coronary imaging have introduced a range of technical strategies designed to mitigate the effects of respiratory motion. Navigator-based methods, self-gating, and fiber optic respiratory monitoring technologies are crucial in detecting and compensating for motion artifacts. These innovations significantly enhance image quality and reliability, ensuring more accurate assessments in coronary imaging and improving patient outcomes in cardiovascular diagnostics.

#### 3.1.1 Respiratory Navigator Technology

Respiratory navigator technology is pivotal in CMRA because it mitigates respiratory motion artifacts. Moreover, respiratory navigator technology tracks diaphragmatic positions in real-time during image acquisition, ensuring that data are collected at consistent points throughout the respiratory cycle [6]. Respiratory navigator technology supports free-breathing protocols by focusing on specific diaphragm positions, allowing for longer acquisition times that result in improved spatial resolution. Despite its advantages, this technology has inherent limitations, including longer scan durations [13]. Additionally, individuals with irregular breathing patterns may experience lower efficiency in data acquisition, as the technology relies on predictable diaphragmatic movement for optimal performance [14]. Recent advancements have sought to improve respiratory navigation technology. Indeed, Plein *et al*. [15] developed an adaptive approach combining individualized cardiac acquisition windows with motion-based respiratory gating. This method achieved significant scan time reductions (2.3-fold for right coronary artery and 2.2-fold for left coronary artery) compared to conventional fixed-window acquisition, while maintaining comparable image quality. Moreover, the technique demonstrated robust diagnostic performance, with 74.3% sensitivity and 88.2% specificity in detecting coronary artery stenosis.

#### 3.1.2 Self-gated Technology

Self-gated technology represents a significant advancement in cardiac MRI, driven by the necessity to reduce reliance on external monitoring devices inherent in traditional imaging approaches. Unlike conventional methods, self-gated technology extracts motion-related information directly from raw data, thereby obviating the need for external devices and markedly enhancing operational convenience and patient comfort [6]. The core process in this method employs sophisticated signal processing algorithms to identify periodic respiratory and cardiac changes from continuously acquired data streams. Meanwhile, during post-processing, the optimal data are selected for image reconstruction to reduce motion artifacts [16].

The primary advantages of self-gated technology include simplified scanning protocols and enhanced patient compliance. Furthermore, the strong ability of self-gated technology to adjust to irregular breathing patterns makes this technique especially beneficial for patients with unique needs, such as children, older populations, and those with respiratory diseases. Stehning *et al*. [17] demonstrated the efficacy of this approach through a self-navigated three-dimensional (3D) radial balanced steady-state free precession (SSFP) whole-heart CMRA technique under free-breathing conditions. This method extracts respiratory signals directly from collected echoes for rigid body motion correction, eliminating additional navigation pulses and simplifying scan planning. Phantom experiments demonstrated significant improvements in image quality, while preliminary *in vivo* studies showed comparable quality to traditional methods with reduced respiratory artifacts. Azhe *et al*. [14] performed a comparative analysis of diaphragm navigation (dNAV) versus self-navigation (sNAV) CMRA in pediatric congenital coronary artery anomalies (CAAs), reporting that sNAV-CMRA exhibited a superior performance in several metrics: higher success rates (100% vs. 93.8%) and reduced scan times (7.3 vs. 9.1 minutes). Further, although sNAV-CMRA showed slightly lower image quality than dNAV-CMRA, diagnostic accuracy remained equivalent, suggesting that sNAV-CMRA represents a viable alternative imaging strategy.

#### 3.1.3 Fiber Optic Respiratory Monitoring

Fiber optic respiratory monitoring forms an emerging technology for high-precision, real-time respiratory assessment by detecting thoracic motion-induced optical signal variations. These systems primarily utilize light reflection, interference, or diffraction principles to detect minute transmitted light changes corresponding to external physical variations.

Fiber optic sensors offer superior accuracy and resistance to artifacts compared to traditional monitoring techniques, enabling the precise capture of subtle respiratory-induced displacements. Two primary optical sensor technologies dominate this field: Optical interference and fiber bragg grating (FBG) [18]. Interference-based sensors detect external physical quantities by measuring phase differences or interference fringe variations between dual-path light beams [19]. While these sensors excel in detecting subtle thoracic displacements and respiratory pressure fluctuations, their complex structure and environmental sensitivity can limit clinical applicability [20]. Comparatively, FBG sensors operate by reflecting specific wavelengths through optical FBGs, monitoring external strain or temperature-induced wavelength shifts [19]. The compact design of the technology, alongside a resistance to electromagnetic interference and capability for multi-point monitoring, makes this technique ideal for use in MRI environments [21].

Recent developments in FBG technology have opened new avenues in clinical applications, particularly in cardiac monitoring. Nedoma *et al*. [22] deployed an FBG-based cardiac triggering system in a 1.5T MRI setting, achieving statistical consistencies of 95.36% for respiratory and 95.13% for heart rate measurements, within 1.96 standard deviations. The FBG sensors exhibited relative errors below 5%, enhancing image quality compared to traditional ECGs and pulse oximetry. Further optimization in 3T MRI environments yielded relative errors of 4.64% and 4.87% for heart and respiratory rate monitoring [23], respectively, demonstrating superior performance in signal stability and trigger accuracy by FBG sensors over traditional ECG methods. Additionally, Brablik *et al*. [24] highlighted additional advantages, including reduced magnetic field interference, enhanced operational efficiency, and improved patient comfort, underscoring the potential of FBG sensors for widespread clinical implementation, particularly in high-field MRI applications.

### 3.2 Heart Rate on Image Stability

Heart rate is a critical determinant of image quality in CMRA, with coronary arterial motion during cardiac cycles posing significant challenges for high-quality image acquisition. Heart rate characteristics substantially influence both image quality and diagnostic utility. Elevated heart rates (>70 beats per minute) reduce the diastolic phase duration, compromising the optimal imaging window and promoting degraded image quality, increased motion artifacts, and diminished spatial resolution and signal-to-noise ratio. While beta-blockers and similar pharmacological agents are commonly employed to reduce heart rate and extend the diastolic phase, cardiac arrhythmias present additional complexities. This temporal irregularity compromises accurate image acquisition, manifesting as increased image blurring, motion artifacts, and inconsistent coronary segment visualization, characterized by missed and redundantly imaged segments. These challenges have prompted the development of various advanced technical strategies for heart rate management in CMRA.

#### 3.2.1 Adaptive ECG Gating Techniques

ECG gating is a fundamental method for synchronizing data acquisition with cardiac cycles to minimize motion artifacts. While conventional prospective ECG gating is susceptible to heart rate variability, recent adaptive real-time techniques offer dynamic adjustment in acquisition windows to accommodate these fluctuations. Furthermore, several innovative approaches have emerged to address these limitations. Yerly *et al*. [25] introduced a self-gated cardiac dynamic MRI sequence that enhanced the evaluation of coronary endothelial function. This method offers enhanced temporal and spatial resolution, comparable to traditional ECG-gated techniques. Moreover, the self-gating approach eliminates reliance on external triggers, providing a more robust imaging solution. Han *et al*. [26] proposed an integrated self-gating approach utilizing k-space data acquisition and principal component analysis. This innovative technique enables real-time monitoring of cardiac motion, achieving a remarkable 100% detection accuracy. Such advancements are particularly beneficial for patients with arrhythmias, where traditional gating methods may fail due to irregular heart rhythms. Thus, by harnessing real-time data, clinicians can obtain clearer and more accurate images of cardiac function. In addition, Bonanno *et al*. [27] introduced a self-gated golden-angle spiral dynamic MRI method to remove the ECG dependency in assessing coronary endothelial function. These alterations enhanced the imaging efficiency of this method, and the image quality and vessel edge were as good as or even better than those of conventional ECG-gated imaging methods when the heart rate was raised or the ECG signal was unstable.

#### 3.2.2 Image-based Navigator

Determining the trigger delay to position the acquisition window during the static phase in the cardiac cycle is crucial for minimizing cardiac motion in CMRA. However, the optimal trigger delay and duration of the acquisition window are still highly dependent on operator input, whereby an inaccurate determination of the trigger delay and acquisition window can increase cardiac motion during image acquisition, leading to reduced image quality. The image-based navigator (iNAV) addresses this issue by automating the motion compensation process. Using low-resolution images to track real-time cardiac motion, the iNAV precisely synchronizes the timing of high-resolution image acquisition with the minimal motion phase of the heart, effectively reducing the need for manual adjustments and improving consistency, reproducibility, and overall image quality. The iNAV has shown substantial improvements in the quality of CMRA. Henningsson *et al*. [28, 29] systematically evaluated the application and performance of iNAV CMRA in multiple studies. In the first study, Henningsson *et al*. [28] compared iNAV CMRA with the traditional diaphragm navigation technique and found that the scan time was significantly reduced (7 min 57 s vs. 9 min 15 s) when using the iNAV, while the vessel sharpness and image quality of the right coronary artery and left anterior descending artery were improved. The iNAV technique also simplified scan planning, resulting in higher scanning efficiency. In a subsequent study, Henningsson *et al*. [29] evaluated the diagnostic performance of iNAV CMRA in patients with coronary artery disease, demonstrating a sensitivity and specificity of 86% and 83%, respectively, with diagnostic image quality achieved in 98% of proximal coronary artery segments.

### 3.3 Cardiac Metal Implants

Metal implants present critical challenges in CMRA, affecting both image quality and patient safety. The increasing prevalence of cardiac implants, including pacemakers, defibrillators, coronary stents, and artificial heart valves, necessitates specific considerations during imaging procedures.

The impact of metal implants on CMRA manifests in several critical aspects. First, the metal implants generate magnetic susceptibility artifacts in strong magnetic fields, causing signal loss and geometric distortion that can obscure surrounding tissue anatomy [30]. Coronary stents exemplify this challenge, creating signal voids that complicate in-stent restenosis assessment. Second, metal implants can disrupt radiofrequency fields, leading to image nonuniformity and potential localized heating. This disruption not only compromises image quality but also risks thermal injury. Additionally, electromagnetic interference may disturb the functionality of electronic implants, such as pacemakers, raising significant safety concerns.

Several strategies address these challenges [31, 32, 33]. First, it is essential to thoroughly understand the type, material, and location of the implant and review its MRI compatibility before CMRA. Second, technical solutions include optimizing scan parameters, such as reducing echo time, lowering flip angles, and increasing receiver bandwidth, to minimize metal artifacts [31, 34]. Furthermore, advanced metal artifact reduction techniques, such as slice encoding for metal artifact correction and view angle tilting, have proven effective in improving image quality near implants [32, 35, 36]. The development of MRI-conditional implants has expanded CMRA accessibility; however, alternative imaging modalities, such as CCTA or echocardiography, remain necessary for contraindicated cases.

### 3.4 Pharmacological Factors on Imaging

The use of adjunctive medications represents a critical yet frequently underappreciated factor affecting the quality of CMRA. Various pharmacological agents can influence CMRA image quality and diagnostic efficacy through distinct mechanisms. The influence of adjunctive medications on CMRA imaging is complex, encompassing aspects such as image quality, patient safety, and imaging tolerability. Clinicians must possess an in-depth understanding of the pharmacodynamics and pharmacokinetics of these agents, as well as their potential implications for imaging, to tailor medication protocols according to the individual clinical profile of each patient.

#### 3.4.1 Adenosine

Adenosine is a pharmacological agent frequently used in CMRA for coronary functional and stress perfusion imaging of the coronary arteries. The cardiac workload increases, and the myocardial blood flow is enhanced through adenosine-induced coronary vasodilation. These characteristics are beneficial for identifying ischemic areas, especially in patients without abnormalities at rest [37]. Heer *et al*. [38] have recently investigated adenosine for non-contrast CMRA paired with stress perfusion MRI. Heer *et al*. [38] achieved good diagnostic accuracy (91.5% for CAD) for myocardial perfusion using 1.5T non-contrast CMRA, highlighting the importance of adenosine in clinical practice. However, the use of adenosine is not without risks since the agent can cause side effects, including hypotension, bradycardia, and respiratory distress, particularly in individuals with underlying health conditions such as asthma or chronic obstructive pulmonary disease [39]. Therefore, adenosine must be administered under careful medical supervision to mitigate these risks.

#### 3.4.2 Beta-blockers

Beta-blockers are another commonly used pharmacologic agent in CMRA for patients with elevated heart rates [40]. These agents slow the heart rate and prolong the diastole, creating an additional resting period and dramatically improving the overall coronary imaging efficiency. This approach yields substantial advantages for patients with heart rates greater than 80 beats/min; image resolution and the signal-to-noise ratio (SNR) can be dramatically improved with this approach [41]. However, since beta-blockers can induce bradycardia and respiratory distress in patients with bronchial asthma, severe heart failure, or cardiac conduction abnormalities, these agents should be used cautiously for these patients [42].

#### 3.4.3 Nitrates

Nitrates are also employed in CMRA to enhance coronary artery visualization. Here, nitrates induce vasodilation to significantly improve the depiction of small-diameter coronary segments, especially in vascular stenosis. Heer *et al*. [43] demonstrated that sublingual administration of nitroglycerin markedly increased coronary visibility, elevating the detection rate of stenosis greater than 50% from 61.8% to 79.4%. Nitroglycerin enhances the diagnostic capability of MRCA for CAD by increasing vessel diameter and extending the visible vessel length. However, nitrates can also precipitate reflex tachycardia, resulting in heart rate fluctuations that may compromise imaging stability [44]. Consequently, beta-blockers are frequently co-administered to mitigate the nitrate-mediated increase in heart rate, thereby optimizing imaging quality.

## 4. Technical Factors on Imaging Quality

### 4.1 Static Magnetic Field Strength

Static magnetic field strength fundamentally influences CMRA quality. Modern systems have evolved from 0.5T and 1.5T to 3T in clinical practice, with 7T systems emerging in research settings, significantly impacting image quality and clinical applications.

Higher field strengths provide enhanced SNR, which theoretically increases linearly with field strength [45]. For instance, upgrading from 1.5T to 3T can theoretically double the SNR. Meanwhile, higher SNRs yield clearer and more detailed coronary artery images, crucial for visualizing small coronary branches and detailed vascular structures [46]. This improvement facilitates more accurate assessment of coronary stenosis, plaque characteristics, and wall structures. Di Leo *et al*. [47] conducted a systematic review and meta-analysis of the diagnostic performance of CMRA in detecting CAD, showing that the specificity of 3T CMRA (83%) was higher than that of 1.5T (68%), further supporting the advantages of higher field strength in coronary imaging. Recent advances in ultra-high-field imaging have shown promising results. Van Elderen *et al*. [48, 49] showed that 7T MRI provides enhanced visualization of the right coronary artery compared to 3T systems, particularly in vessel edge sharpness and blood-to-tissue contrast, enabling better detection of early atherosclerotic lesions.

However, high-field CMRA presents several technical challenges. Firstly, radio frequency inhomogeneity increases with field strength, causing image brightness variations and compromising quantitative analysis [50]. Secondly, specific absorption rate (SAR) limitations become more restrictive, as the SAR increases quadratically with field strength, potentially constraining certain imaging sequences [51]. Thirdly, magnetic susceptibility artifacts intensify, particularly at tissue–air interfaces, affecting coronary vessel visualization [52]. Fourthly, B0 inhomogeneity becomes more pronounced, impacting large field-of-view imaging and fat suppression techniques [53]. Fifthly, ECG signal interference increases, complicating cardiac synchronization [54]. Nonetheless, these limitations are being mitigated by ongoing technological advancements in high-field CMRA regarding image reconstruction, artifact correction, and quantification.

### 4.2 Imaging Sequences

The selection of imaging sequences in CMRA critically influences image quality and spatiotemporal resolution. Field strength fundamentally determines sequence selection, with SSFP sequences optimized for 1.5T systems and gradient echo (GRE) sequences better suited for 3T applications.

SSFP sequences are widely used for their high SNR and good tissue contrast in 1.5T MRI, compared to surrounding static tissues. SSFP sequences can improve the contrast between blood and surrounding static tissues by balancing the transverse magnetization vector within the plane of interest to the equilibrium state, which facilitates the visualization of coronary details and results in good image quality at 1.5T [55]. Since the SSFP sequence is vulnerable to magnetic field homogeneity, the susceptibility to inhomogeneity and off-resonance effects at 3T causes signal loss and artifacts [51]. Therefore, SSFP sequences are considered more reliable for 1.5T systems.

GRE sequences show improved performance at 3T due to their property of being less sensitive to magnetic susceptibility variations. Meanwhile, by removing the requirement for continuous maintenance of transverse magnetization, GRE sequences exhibit decreased sensitivity to field inhomogeneities and help minimize banding artifacts [55]. With the SNR increase at 3T, GRE sequences can improve spatial resolution, particularly when used with contrast agents to improve the vascular contrast. GRE sequences frequently incorporate spin preparation pulses (T2 preparation or inversion recovery) to suppress background tissue signals and enhance coronary contrast. Furthermore, the T2* sensitivity of GRE is useful because it allows for the detection of pathological conditions, such as hemorrhage and fat deposition, which may not be readily apparent on other sequences.

### 4.3 Contrast Agents

Contrast agent use in CMRA is beneficial since these agents can enhance the contrast between the coronary arteries and the surrounding myocardium [6]; there are several classes of contrast agents that are available today, such as conventional gadolinium containing agents, blood pool agents and ultrasmall superparamagnetic iron oxide (USPIO) particles [56].

#### 4.3.1 Gadolinium-based Contrast Agents

Gadolinium-based contrast agents are still predominantly used in CMRA due to their advantages. These agents increase blood signal intensity by shortening T1 relaxation time, enhancing arterial–tissue contrast [56]. Further, the efficacy of these agents is particularly notable at high magnetic field strengths (e.g., 3T), where challenges such as banding artifacts and B1 inhomogeneity can limit the use of SSFP sequences. Consequently, GRE sequences combined with gadolinium-based agents optimize coronary artery visualization, significantly improving the SNR and image quality for detecting small vascular branches and complex lesions [57]. Research by Kato *et al*. [58] demonstrated that contrast-enhanced 3T whole-heart coronary angiography (WHCA) offers superior specificity to non-contrast 1.5T WHCA. However, gadolinium-based agents pose potential risks for patients with renal dysfunction, notably nephrogenic systemic fibrosis [59]. Clinicians must carefully evaluate contrast agent administration in renally impaired patients, potentially utilizing low-dose or alternative contrast protocols.

#### 4.3.2 Blood Pool Agents

Blood pool agents (BPAs) represent an advanced contrast mechanism in CMRA, offering unique advantages over conventional contrast agents. These agents enable prolonged intravascular retention and extended contrast enhancement by binding to plasma proteins [60, 61]. This property renders BPAs especially effective for evaluating complex vascular anatomy and coronary artery lesions, making them well-suited for scenarios requiring multiple or delayed imaging sessions. Gadofosveset trisodium is an example of a BPA that promotes clear improvements in both image quality and ability to impact clinical outcomes. In a landmark study, improvements in coronary images allowed for an increase in evaluable coronary segments from 79% to 89% and an increase in diagnostic specificity from 68.3% to 80.2% [60]. Thus, these agents show great potential to transform cardiovascular MRI for improved diagnostics. However, while BPAs have many potential advantages over current contrast agents, these agents also pose significant clinical and market risks. Gadofosveset trisodium was discontinued in 2017 due to low demand and high costs, limiting clinical use of BPAs. Additional limitations for BPAs include the impact on clinical use of long scan times, which may compromise patient compliance, and concerns regarding allergic reactions.

#### 4.3.3 Ultrasmall Superparamagnetic Iron Oxide Particles

USPIO particles are a new type of contrast agent for MRI, and are especially promising for CMRA. Moreover, USPIO particles exhibit many advantages in the cardiovascular field owing to the extremely small size and long blood circulation time, including stability and long-lasting contrast enhancement, which are essential for both high-spatial resolution and long acquisition time courses [61, 62]. Recent studies have highlighted the significant potential of USPIO particles. Piccini *et al*. [63] illustrated the potential of the agent by applying respiratory self-navigation with ferumoxytol to obtain continuous contrast enhancement and good visualization of the stable vessel during different respiratory phases. The long-lasting contrast properties of USPIO particles also made the distinct delineation of multiple coronary branches possible even with significantly large motion artifacts. Dong *et al*. [64] evaluated the diagnostic performance and safety of the USPIO-based contrast agent ferumoxytol in patients with CAD. Dong *et al*. [64] found that the segment-level sensitivity and specificity of ferumoxytol-enhanced CMRA for coronary stenosis were 92.3% and 96.7%, respectively, demonstrating good ability to visualize small vessels and high accuracy of coronary vessel segmentation and coronary lesion detection. Furthermore, ferumoxytol improved safety in patients with renal impairment compared to the conventional gadolinium-based agent. There were no serious adverse events in renal impairment patients during the three-month follow-up, which positioned the USPIO particles as the ideal substitute for contraindicated patients to use gadolinium- or iodine-based contrast agents.

## 5. Advances in Accelerated Imaging Techniques

Due to its traditional acquisition process, CMRA is time-consuming and very vulnerable to cardiac motion, respiratory motion, and blood flow fields, which result in the appearance of artifacts or low image quality and further impair the reliability of the diagnosis. Therefore, reducing the acquisition time and maintaining the image quality remain key issues in clinical applications. Certainly, minimizing these limitations is also important since these actions can improve the comfort of patients and reduce the impact of motion artifacts. Many accelerated acquisition methods have been developed, including parallel imaging (PI), compressed sensitivity encoding (CS), and deep learning methods.

### 5.1 Parallel Imaging Technology

PI is a commonly used accelerated imaging approach in CMRA. PI implements a multi-coil receiving system, which can accelerate the data acquisition at the expense of a decreased SNR [65]. The most commonly used parallel imaging methods, such as sensitivity encoding (SENSE) and generalized autocalibrating partial parallel acquisition (GRAPPA), have been applied in CMRA. SENSE reconstructs undersampled data in the image domain with coil sensitivity information, and GRAPPA applies an interpolation approach in k-space to reconstruct the full data [66, 67]. The merits of PI are that this technology can greatly reduce the time required for imaging and, therefore, can partially correct for respiratory motion blurring. However, the reduced SNR cannot be ignored and is a limiting factor. Hence, a compromise is always required between the acceleration rate and SNR.

### 5.2 Compressed SENSE Technology

CS is an accelerated imaging technique predicated on signal sparsity, which reconstructs images from undersampled data by leveraging the sparse representation of the image domain [68]. CS is particularly advantageous for cardiac imaging, as cardiac images exhibit high sparsity in specific transform domains, such as wavelet or Fourier domains. Moreover, CS reconstructs the original image by solving a nonlinear optimization problem, with commonly employed CS algorithms including regularized least squares and gradient projection methods [69]. In contrast to parallel imaging, CS emphasizes exploiting the inherent properties of the image, facilitating effective image reconstruction even under conditions of a reduced SNR. Zhang *et al*. [67] and Tian *et al*. [70] conducted comparative analyses of CS and conventional SENSE techniques in CMRA. The findings of these two studies indicated that CS substantially reduced the scan time for whole-heart CMRA and maintained or enhanced image quality, underscoring the superiority of CS in coronary imaging.

### 5.3 Deep Learning-based Methods

Several advanced methods have recently been proposed to enhance accelerated image acquisition further and mitigate noise to optimize image quality, including incorporating deep learning-based image reconstruction algorithms. These approaches have effectively addressed challenges such as SNR degradation and prolonged reconstruction times. Hence, training deep learning techniques on extensive datasets of high-quality images can promote understanding of complex imaging features, such as edge sharpness and texture details, which contribute to improving reconstruction accuracy and preserving fine anatomical structures in undersampled data. Prominent deep learning reconstruction models include U-Net, generative adversarial networks, and convolutional neural networks [71]. Meanwhile, previous studies [72, 73, 74] have demonstrated the promising opportunities of deep learning in accelerating imaging, improving image quality, and cardiovascular disease diagnosis. Yokota *et al*. [72] applied deep learning reconstruction techniques and greatly enhanced CNR and vessel clarity in high-resolution non-contrast MRA. Moreover, their method yielded excellent results in imaging distal coronary arteries. Wu *et al*. [73, 74] applied deep learning-based compressed sensing (DL-CS) in non-contrast CMRA. These studies show that these methods provide higher image quality, diagnostic performance, significantly reduced scan times, and notably improved sensitivity, specificity, SNR, and CNR. Furthermore, beyond acceleration, deep learning methods have recently demonstrated the potential to reduce contrast agent doses by 80% in CMRA while maintaining diagnostic accuracy. For example, Montalt-Tordera *et al*. [75] applied a residual U-Net trained on synthetic low-dose data to reconstruct near-full-dose images, where the achieved SNR (56.5 vs. 53.6) and CNR (52.4 vs. 50.0) were very close to full dose. The method also achieved 88.2% sensitivity and 96.0% specificity for detecting vascular abnormalities, a crucial first step toward reducing contrast agent doses in safer protocols for vulnerable populations that require longitudinal tracking. Most clinical sites will comprise deep learning reconstruction servers connected to the existing MR scanners via the digital imaging and communications in medicine interface for clinical deployment of these deep learning reconstruction methods. The implementation of deep learning in clinical MR workflows consists of three implementation phases: (1) An initial benchmarking phase to characterize DL-reconstruction images compared with conventional techniques across various cardiac pathologies, (2) training courses for radiologists to appreciate the typical features and potential artifacts in deep learning reconstructions, and (3) internal quality control studies to monitor the reconstruction performance. Meanwhile, implementing techniques such as DL-CS and low-dose contrast enhancement models would require multidisciplinary (radiology and information technology) cooperation to manage the data handling issues and integrate the new deep learning workflows into the existing picture archiving and communication systems. These new reconstruction techniques can be applied as alternative methods to the currently used conventional reconstruction techniques. However, challenges remain for deep learning methods, such as model generalization, dependence on large-scale labeled data, and model interpretability in clinical applications.

## 6. Future Perspectives

Non-contrast CMRA shows great potential for noninvasive CAD assessment; however, several issues must be solved to enable clinical adoption. First and foremost, respiratory and cardiac motion artifacts continue to impair image quality despite advances in the navigator technique. To overcome this obstacle, future developments should likely incorporate artificial intelligence (AI)-driven motion prediction algorithms alongside hybrid approaches that integrate multiple compensation strategies, ultimately reducing dependency on patient cooperation. Moreover, the persistent trade-off between spatial resolution and scan duration necessitates innovative solutions such as ultra-high-field MRI systems and advanced reconstruction methods to improve SNRs without extending acquisition times. The variable performance of CMRA across diverse patient populations also calls for patient-specific protocol optimization through AI-powered tools, enhanced metal artifact reduction techniques, and specialized sequence adaptations for challenging cohorts, including pediatric and geriatric patients. From a practical standpoint, clinical integration hinges on streamlining workflow processes through cloud-based AI platforms for automated image analysis and establishing consensus guidelines to improve reproducibility across institutions. Furthermore, despite encouraging meta-analyses, stronger clinical validation through prospective multi-center trials comparing CMRA directly to established reference standards remains essential, particularly for high-risk populations who would benefit most from noninvasive alternatives. Lastly, the advancement of non-contrast CMRA depends on interdisciplinary collaboration among engineers, clinicians, and computational scientists who can systematically address technical limitations while ensuring clinical relevance, transforming CMRA from a specialized tool into a mainstream diagnostic modality and a cornerstone of modern cardiovascular imaging.

## 7. Conclusions

CMRA, as a noninvasive, high-resolution imaging modality, shows significant promise in the early diagnosis of CAD. This paper reviewed key factors influencing CMRA image quality, including physiological and technical aspects. These factors are crucial to the CMRA process, affecting both image quality and diagnostic accuracy. With ongoing innovation, CMRA is set to play a significant role in CAD diagnoses, providing reliable and efficient diagnostic tools for clinical use.
